# Phenolic Compounds Contribution to Portuguese Propolis Anti-Melanoma Activity

**DOI:** 10.3390/molecules28073107

**Published:** 2023-03-30

**Authors:** Ana Rita Caetano, Rafaela Dias Oliveira, Sónia Pires Celeiro, Ana Sofia Freitas, Susana M. Cardoso, M. Sameiro T. Gonçalves, Fátima Baltazar, Cristina Almeida-Aguiar

**Affiliations:** 1 Department of Biology, School of Sciences, University of Minho, Campus of Gualtar, 4710-057 Braga, Portugal; 2 Centre of Molecular and Environmental Biology (CBMA), University of Minho, Campus of Gualtar, 4710-057 Braga, Portugal; 3 Life and Health Sciences Research Institute (ICVS), School of Medicine, University of Minho, Campus of Gualtar, 4710-057 Braga, Portugal; 4 ICVS/3B’s-PT Government Associate Laboratory, 4710-057 Braga, Portugal; 5 LAQV-REQUIMTE, Department of Chemistry, University of Aveiro, 3810-193 Aveiro, Portugal; 6 Centre of Chemistry (CQ/UM), Department of Chemistry, University of Minho, Campus of Gualtar, 4710-057 Braga, Portugal

**Keywords:** Portuguese propolis, natural products, melanoma, antitumoral activity, fractionation, phenolic compounds

## Abstract

Melanoma is the deadliest type of skin cancer, with about 61,000 deaths annually worldwide. Late diagnosis increases mortality rates due to melanoma’s capacity to metastasise rapidly and patients’ resistance to the available conventional therapies. Consequently, the interest in natural products as a strategy for drug discovery has been emerging. Propolis, a natural product produced by bees, has several biological properties, including anticancer effects. Propolis from Gerês is one of the most studied Portuguese propolis. Our group has previously demonstrated that an ethanol extract of Gerês propolis collected in 2018 (G18.EE) and its fractions (*n*-hexane, ethyl acetate, and *n*-butanol) decrease melanoma cell viability. Out of all the fractions, G18.EE-*n*-BuOH showed the highest potential as a melanoma pharmacological therapy. Thus, in this work, G18.EE-*n*-BuOH was fractioned into 17 subfractions whose effect was evaluated in A375 *BRAF*-mutated melanoma cells. The subfractions with the highest cytotoxic activity were analysed by UPLC-DAD-ESI/MS^n^ in an attempt to understand which phenolic compounds could account for the anti-melanoma activity. The compounds identified are typical of the Gerês propolis, and some of them have already been linked with antitumor effectiveness. These results reaffirm that propolis compounds can be a source of new drugs and the isolation of compounds could allow its use in traditional medicine.

## 1. Introduction

Cancer is currently the second-leading cause of death worldwide, responsible for around 10 million deaths in 2020 [1]. Melanoma is the deadliest skin cancer type, causing about 61,000 deaths per year around the world [2,3]. If diagnosed at an early stage, it can be removed by surgery, improving the patient’s survival rate. However, late diagnosis increases mortality rates due to its high capacity to metastasize, making treatment much more challenging [4,5]. One of the most recent options for melanoma treatment is targeted therapy, being the combination of *BRAF* (V-RAF murine sarcoma viral oncogene homolog B) and *MEK* (mitogen-activated protein kinase kinase) inhibitors used to enhance survival in *BRAF*-mutated patients [6,7,8]. The melanoma genome is highly mutated, with the most common mutation associated with the *BRAF* gene, a part of the mitogen-activated protein kinase (MAPK) signalling pathway [9,10,11]. *BRAF* mutation over-activates this pathway, leading to tumour aggressiveness and progression [12,13]. Approximately 40% to 60% of melanoma patients have activated *BRAF* mutations [14] and the most common mutation is found at amino acid 600, in which valine is replaced by glutamic acid (*BRAF* ^V600E^) [15,16]. However, the major problem with these targeted therapies is the development of multiple resistance mechanisms [17,18,19].

Humanity is always searching for novel treatments for diseases, of which natural products remain one of the most popular sources [20,21,22]. In recent years, the tendency to look at natural products as a strategy for drug discovery has re-emerged [23]. It is estimated that about a third of the drugs approved by the FDA (Food and Drug Administration) in the last 20 years are based on natural products [24]. Propolis is a natural product produced by honeybees, namely *Apis mellifera*, that collect resins from resinous sprouts and exudates of plants and mix this resin with salivary *β*-glycosidase, pollen, and waxes to form the final product [25,26,27,28]. Propolis has been used as a natural remedy over the years due to its exceptional pharmacological and biological properties, which include antibacterial, antitumour, antifungal, anti-protozoal, anti-viral, antioxidant, anti-inflammatory, hepato-protective, cardioprotective, anti-neurodegenerative, antituberculosis, local-anaesthetic, immunostimulant, cytotoxic, antiaging, or cicatrizing properties [29,30,31,32]. However, the difficulty in turning propolis into a pharmaceutical product is due to the complicated standardization of its chemical composition [33,34,35]. Indeed, its chemical components are highly variable depending on distinct factors, including the bee’s species, the plant source, the geographical location, the climatic conditions, the time of collection, and the harvesting method [36,37]. About 800 compounds already have been identified in propolis, but new compounds are expected to be identified [30]. The main classes of propolis bioactive compounds include phenolic acids (cinnamic and caffeic acids) and their esters, flavonoids (flavones, flavanones, flavonols, and dihydroflavonols chalcones), and terpenes [28,38,39].

Propolis from Gerês, one of the most studied Portuguese propolis [40,41,42,43,44,45,46], displays high antioxidant and antimicrobial activities, regardless of the harvesting year [40]. An ethanol extract of propolis from Gerês collected in 2018 (G18.EE) and its three fractions—ethyl acetate (G18.EE-EtOAc), *n*-hexane (G18.EE-*n*-hexane), and *n*-butanol (G18.EE-*n*-BuOH)—promoted apoptosis mediated by ROS (reactive oxygen species) in melanoma, thus demonstrating to be cytotoxic to *BRAF*-mutated melanoma cell lines. G18.EE-*n*-BuOH emerged as the fraction with the highest activity among the tested samples. This result suggests that G18.EE-*n*-BuOH can be an interesting melanoma therapeutic source [43]. In this way, G18.EE-*n*-BuOH was fractioned to separate its components in subfractions according to their polarity, and the cytotoxic effect of the obtained subfractions was assessed in A375—a *BRAF*-mutated melanoma cell line. The composition of the most active subfractions was analysed in an effort to understand which phenolic compounds could account for the anti-melanoma activity.

## 2. Results

### 2.1. Cell Viability Assay

**Figure 1:**
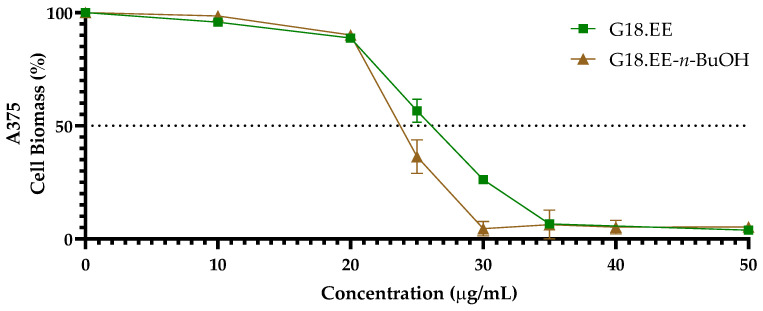
Effect of G18.EE and G18.EE-*n*-BuOH on total cell biomass of A375 melanoma cell. Cells were treated with a range of concentrations (10 to 50 µg/mL) of G18.EE and G18.EE-*n*-BuOH for 72 h to determine the IC_50_ concentrations. Data were normalized for total biomass. Results are presented as mean ± SD (n ≥ 3).

The cytotoxic effect of G18.EE and G18.EE-*n*-BuOH was evaluated against the A375 *BRAF*-mutated human melanoma cell line through the sulforhodamine B (SRB) assay. The tested samples effectively decreased the A375 cell biomass in a dose-dependent manner, and the values of IC_50_ (Table 1) were calculated through the curve shown in Figure 1. It is possible to observe that A375 cells seem to be more sensitive to G18.EE-*n*-BuOH compared to G18.EE. Based on these results, it was decided to proceed with the G18-*n*-BuOH fractionation.

### 2.2. Subfractionation of G18.EE-n-BuOH Fraction

**Figure 2:**
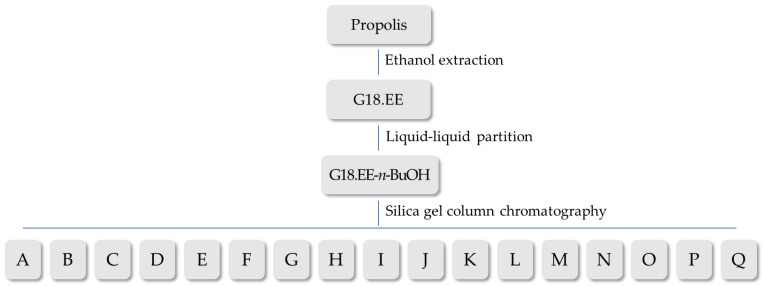
Methodologies used to obtain G18.EE-*n*-BuOH and the 17 subfractions resulting from its fractionation.

The G18.EE was partitioned into four fractions (G18.EE-EtOAc, G18.EE-*n*-hexane, G18.EE-*n*-BuOH, and G18.EE-water) through a liquid–liquid partition. The G18.EE-*n*-BuOH was further fractionated in a silica gel column chromatography that separates the compounds based on their polarity, in order to obtain subfractions with less complex chemical composition. This process results in 17 subfractions (Figure 2).

### 2.3. Cytotoxic Effect of G18.EE-n-BuOH Subfractions

**Figure 3:**
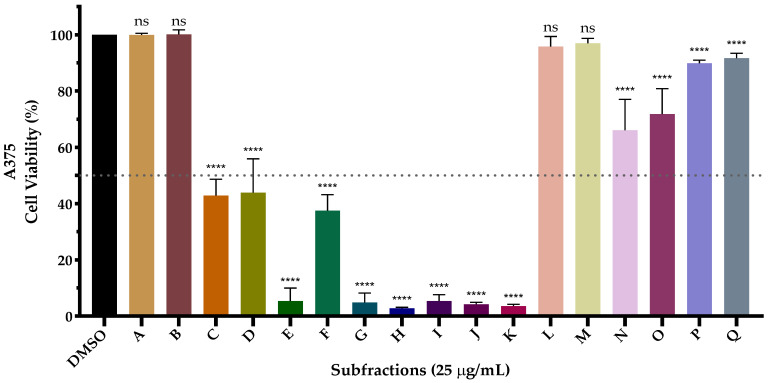
Effect of 25 μg/mL of each subfraction on A375 cell biomass. Cell biomass was measured at 72 h by SRB assay after treatment with the 17 subfractions. Data were normalized to the control condition. The results are presented as mean ± SD (n ≥ 3). The statistical differences are in regard to the control, where “ns” means not significant and **** *p* < 0.0001.

The cytotoxic capacity of all subfractions was tested in the A375 melanoma cell line at 25 μg/mL concentration, corresponding to an intermediate concentration between IC_50_ obtained for both G18.EE and G18.EE-*n*-BuOH (Table 1). The subfractions exhibited different effects on A375 cell viability (Figure 3) The most active subfractions were E, G, H, I, J, and K, while subfractions C, D, and F demonstrated behavior similar to the fraction they were obtained from (G18.EE-*n*-BuOH). The remaining subfractions had a less noticeable effect as they are less cytotoxic than the upstream fraction.

### 2.4. Chemical Analysis of the Subfractions

**Figure 4:**
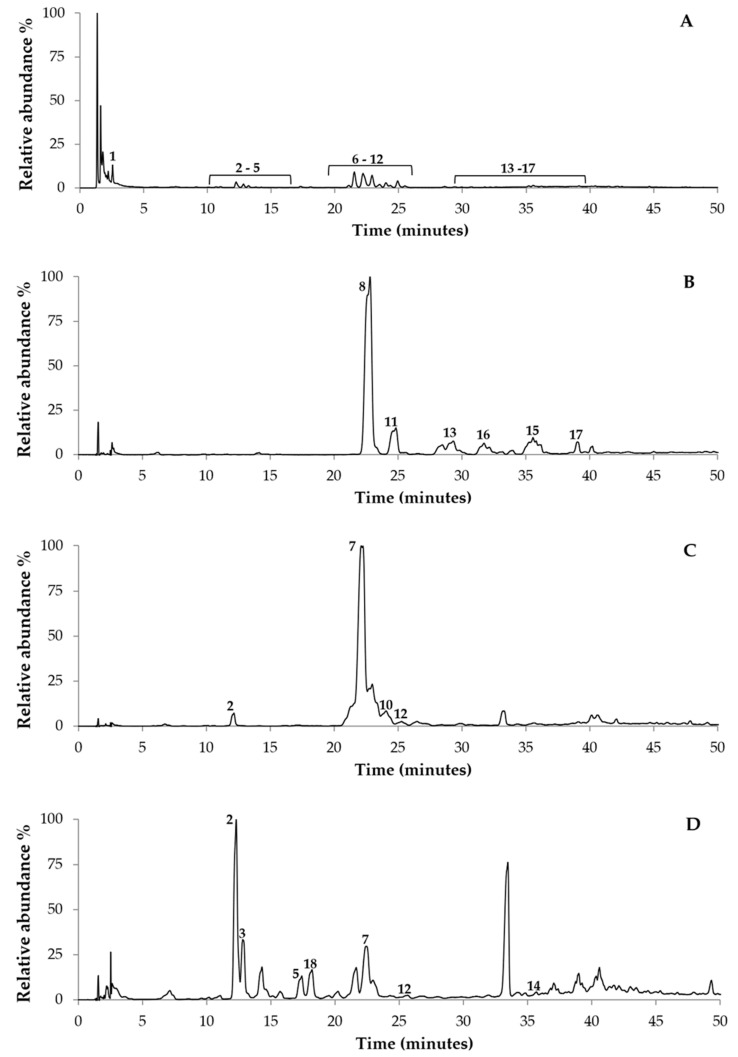
Chromatographic profile of (**A**) G18.EE-*n*-BuOH; (**B**) subfraction E; (**C**) subfraction I, and (**D**) subfraction K obtained by UHPLC-DAD-ESI/MS^n^ (280 nm). Each peak numbered in the figure represents a different compound identified in Table 2.

The phenolic compounds of subfractions E, G, H, I, J, and K were analysed by UPLC-DAD-ESI/MS^n^ as they demonstrate to be the most cytotoxic subfractions against the A375 melanoma cell line. Figure 4 shows the chromatographic profiles at 280 nm for G18.EE-*n*-BuOH and subfractions E, I, and K, and the phenolic composition is detailed in Table 2. Subfractions G, H, and J had chromatographic peaks not defined, probably due to the fact that phenolic compounds are present in very low amounts, not allowing the detection and identification of this type of compound in these subfractions.

## 3. Discussion

Propolis has proven antitumor activity against several types of cancer [42,47,48,49,50,51,52] and, although scarce, some researchers demonstrated its anti-melanoma activity [43,53,54]. Some therapeutic options already exist for melanoma, but their effectiveness is still limited, mainly due to acquired resistance [55]. Thus, the investigation of propolis activity in this type of cancer is important due to the need for new treatments and sources of therapeutic agents [56].

The results mentioned above demonstrated that G18.EE and G18.EE-*n*-BuOH are capable of decreasing A375 cell viability (Table 1, Figure 1). Previous studies had already described the cytotoxic effect of propolis on melanoma. For example, a Chinese propolis ethanol extract had an anti-proliferative effect on A375 (IC_50_ = 112 µg/mL) [57]. The cytotoxic effect of the water extract of propolis was also tested in this cell line, with an IC_50_ value of 353 µg/mL [58]. These latest IC_50_ values for propolis are much higher than the one obtained in this work for Gerês propolis against the A375 cell line (Table 1). An ethanol extract of propolis from Poland was demonstrated to decrease the viability of Me45 melanoma cells [59], as well as Dutch propolis isolated compounds (cinnamic acid derivatives, flavonoids and glycerol derivatives) in murine B16-BL6 melanoma [60]. The hydroalcoholic extracts (70%) of two types of Brazilian stingless bee propolis (Tubuna and Mandaçaia) have a cytotoxic effect in SK-MEL-28 (99 and 35 µg/mL reducing cell number by 50%, for Tubuna and Mandaçaia, respectively) [54]. The ethanol extract of Algerian propolis, as well as compounds isolated from green propolis (namely baccarin and p-coumaric acid), decreased murine melanoma tumour progression [61,62]. In general, Gerês propolis extract and its butanol fraction show lower IC_50_ values (Table 1) than those described in the literature for propolis effects in melanoma, and therefore appear to be more active.

The anticancer effect of G18.EE and its fractions have previously been reported, proving, together with the results described in this work, the higher potential of Gerês propolis for cancer treatment. The cytotoxicity of Gerês propolis (G18.EE) and its four fractions: EtOAc, *n*-hexane, *n*-BuOH, and water were evaluated in renal cancer cell lines (A498, 786-O and Caki-2) and non-neoplastic renal cells (HK2). The EtOAc fraction was the most effective as it presented considerable cytotoxic activity (IC_50_ from 0.16 to 30 µg/mL) and a high selectivity index [42]. The effect of this Gerês extract and its fractions was previously described in two *BRAF*-mutated melanoma cell lines. The highest cytotoxic activity was displayed by the *n*-BuOH fraction against both melanoma cell lines (IC_50_ from 8.14 μg/mL to 11.22 μg/mL) [43].

As mentioned, propolis has a highly variable chemical composition, a major barrier to its use in the pharmaceutical industry and traditional medicine [33]. From this perspective, it is important to isolate and identify which compounds or mixtures of compounds allow G18.EE-*n*-BuOH to have this anti-melanoma activity. Since A375 melanoma cells seemed to be more sensitive to G18.EE-*n*-BuOH treatment (Table 1), G18.EE-*n*-BuOH was fractioned, having resulted in 17 subfractions (Figure 2). The cytotoxic effect of these subfractions was evaluated and some of the subfractions have a considerable impact on the A375 cell viability reduction (Figure 3). The most active fractions were E, G, H, I, J, and K, and in these most effective subfractions, compounds with anti-melanoma activity are expected to be present.

The pharmacological properties of propolis are often attributed to its phenolic compounds [35,63]. In light of this, the phenolic compounds of the most active subfractions were analysed by UPLC-DAD-ESI/MS^n^. Our research group has previously reported the chemical composition of G18.EE and its fractions [43]. In general, the compounds found in the subfractions (Table 2) fit well with what has been described for other Gerês propolis samples [40,42,43]. Moreover, some of the compounds identified here are present in other European propolis samples [64]. Indeed, the phenolic compounds identified in G18.EE-*n*-BuOH include phenolic acids such as ellagic acid, and flavonoids such as kaempferol and galangin. Pinobanksin, pinocembrin, chrysin, and acacetin were identified too, belonging to the class of flavanones and dihydroflavonols. Flavones such apigenin were also found.

Other propolis samples with proven cytotoxic effect on melanoma had their composition described. A Chinese propolis sample was also evaluated against the A375 melanoma cell line [57]. For this sample, the IC_50_ concentration was 112 µg/mL [57], whereas for our most active subfractions, at a concentration of 25 µg/mL (about 4.5 times less concentrated), we obtained a cell viability of approximately 5%. We can observe that the Chinese extract and our subfractions have some compounds in common, such as apigenin, pinobanksin, kaempferol, chrysin, galangin, CAPE, pinobanksin 3-*O*-acetate, and pinocembrin [57]. However, we found that, in the subfractions, acacetin and kaempferide are not present in this Chinese propolis sample, and these active compounds may explain the higher cytotoxicity of the subfractions. Moreover, in this Chinese propolis, the most representative compounds are chrysin, pinobanksin 3-*O*-acetate, and pinocembrin [57], the last two being present in our subfraction E. As subfraction E is much more active than Chinese propolis, other compounds present in this subfraction probably contribute to its higher activity. Kubina et al. [59] demonstrated the anti-melanoma effect of a Polish propolis extract sample, describing a cytotoxicity of 12.2 ± 1.77% (which suggests cell viability of approximately 88%) in the Me45 melanoma cell line, for a concentration of 25 µg/mL of extract, seeming to be less active in comparison to our subfractions (Figure 3). The difference between the activities could be justified by the use of the A375 melanoma cell line in our work, while the authors used the Me45 melanoma cell line. Therefore, to confirm the higher cytotoxic activity of our subfractions, it would be necessary to test them in the Me45 cell line. Even so, analyzing the composition of the extract, we noticed that the phenolic compounds present in higher amounts in this extract [59] have not been identified in our subfractions (caffeic acid, benzoic acid, and ferulic acid). However, the compounds present in a lower proportion in this extract, such as kaempferol, galangin, and apigenin, were also identified in our subfractions, which again reinforces the hypothesis that these are the compounds responsible for the high cytotoxic effect presented by the subfractions.

In addition, CAPE, a phenolic acid present in propolis of various geographical origins, is associated with significant chemoprotective and anticancer properties [33,65,66,67], including against melanoma cells [68]. Acacetin identified in subfractions I and K exhibited anticancer effects in diverse types of cancer such as breast cancer, lung cancer, prostate cancer, and colon cancer [69]. The subfraction K appears to be the one that most affects cell viability, so it may be interesting to look at compounds that are only present in this subfraction—apigenin and kaempferol. Apigenin is a flavonoid with documented anticancer activity. This property is associated with multiple biological processes, including induction of cell cycle arrest, activation of cell apoptosis and autophagy, inhibition of cell migration and invasion, and initiation of an immunological response [70,71]. Moreover, kaempferol present in subfraction K proved to have anticancer properties associated with a mechanism that induces cancer cell apoptosis [72]. Regarding all these results, the compounds identified are potential targets for developing new drugs against cancer. The possible synergism between these compounds and their mechanism of action should be further investigated.

## 4. Materials and Methods

### 4.1. Propolis Sample and Preparation of Gerês Propolis Extract

The propolis sample (designated as G18) was obtained in 2018 from an apiary close to the Cávado River, between the villages of Paradela and Sirvozelo, in Montalegre, Gerês, Portugal (41°45′041.62″ N; 7°58′003.34″ W). The G18 propolis extract was prepared with absolute ethanol (Fisher Chemical) to obtain G18.EE, as previously described by Freitas et al. [40]. The ethanol extract (EE) was then dried by solvent evaporation (62% yield [41]) and stored at 4 °C, in the dark, until further use.

### 4.2. Fractionation of G18.EE

Fractionation of G18.EE was performed as previously described [42]. Succinctly, G18.EE (4 g) was dissolved in absolute ethanol (20 mL) and purified water (200 mL). The solution was successively partitioned with *n*-hexane, ethyl acetate (EtOAc), and *n*-butanol (*n*-BuOH) (3 × 400 mL each). This procedure resulted in organic phases that were pooled and dried over sodium anhydrous sulphate and the solvent evaporated under low pressure, at 40 °C, to obtain the G18.EE-*n*-hexane (1.22 g), G18.EE-EtOAc (3.58 g), G18.EE-*n*-BuOH (54.6 mg), and G18.EE-water (5.6 mg) fractions. All G18.EE fractions were stored at 4 °C in the dark until further use and dissolved in dimethyl sulfoxide (DMSO) (Honeywell, Charlotte, NC, USA) to prepare stock solutions for the next experiments.

### 4.3. Subfractionation of G18.EE-n-BuOH Fraction

The G18.EE-*n*-BuOH was further fractionated in silica gel column chromatography. Briefly, silica gel 60 (0.035–0.070 mm; Acrós Organics, Morris Plains, NJ, USA) diluted in *n*-hexane (Fisher Chemical, Waltham, MA, USA) was placed on a glass column. G18.EE-*n*-BuOH (156.6 mg) diluted in ethanol was deposited on top of the column and eluted successively with *n*-hexane, *n*-hexane/EtOAc mixtures of increasing polarity, and finally with mixtures of EtOAc/MeOH also with increasing polarity. The 54 subfractions obtained were analysed by thin-layer chromatography (TLC) (TLC silica gel F254, Merk, Darmstadt, Germany). The developed plates were examined under UV light (254 nm and 366 nm), followed by spraying with anisaldehyde-H_2_SO_4_ (prepared by adding 0.5 mL of anisaldehyde (Acrós Organics) in 10 mL of fluvial acetic acid (PanReac, Chicago, IL, USA), 85 mL of MeOH, and 5 mL of concentrated H_2_SO_4_ (Fisher Chemical) [73] adapted), and heated for 5 min at 100 °C. The fractions were pooled according to similarities in their TLC-chromatographic profile, finally obtaining 17 main subfractions, which were assigned a code from A to Q. After solvent remotion in a Rotavapor under low pressure at 40 °C, followed by nitrogen flow, subfractions were stored at 4 °C, in the dark, until further assays and dissolved in DMSO to prepare stock solutions for further experiments.

### 4.4. Chemical Analysis of the Subfractions: UPLC-DAD-ESI/MS^n^

The subfractions E, G, H, I, J, and K were analysed by UPLC-DAD-ESI/MS^n^ as described [40]. The UPLC-DAD-ESI/MS^n^ analysis was performed on an Ultimate 3000 (Dionex Co., Sunnyvale, CA, USA) apparatus equipped with an ultimate 3000 Diode Array Detector (Dionex Co.) and coupled to a mass spectrometer LTQ XL Linear Ion Trap 2D. The chromatographic system consisted of a quaternary pump, an autosampler, a photodiode-array detector, and an automatic thermostatic column compartment. Analysis was run on a Hypersil Gold (Thermo Scientific, Waltham, MA, USA) C18 column (100 mm length; 2.1 mm i.d.; 1.9 μm particle diameter, end-capped) and its temperature was maintained at 30 °C. The mobile phase was composed of (A) 0.1% of formic acid (*v*/*v*) and acetonitrile (B). The solvent gradient started with 20% of solvent (B), reaching 40% at 25 min, 60% at 35 min, and 90% at 50 min, followed by the return to the initial conditions. The flow rate was 0.1 mL/min, and UV–Vis spectral data for all peaks were accumulated in the range of 200 to 500 nm, while the chromatographic profiles were recorded at 280 and 320 nm. The mass spectrometer used was a Thermo LTQ XL (Thermo Scientific) ion trap MS equipped with an ESI source. Control and data acquisition were carried out with the Thermo Xcalibur Qual Browser data system (Thermo Scientific). Nitrogen above 99% purity was used, and the gas pressure was 520 kPa (75 psi). The instrument was operated in negative-ion mode with ESI needle voltage set at 5.00 kV and an ESI capillary temperature of 275 °C. The full scan covered the mass range from *m*/*z* 100 to 2000. CID–MS/MS and MS^n^ experiments were simultaneously acquired for precursor ions using helium as the collision gas with a collision energy of 25–35 arbitrary units.

### 4.5. Cell Lines and Culture Conditions

The in vitro assays were performed using a human melanoma cell line: A375 (*BRAF* ^V600E^ mutated). This human cell line was established from cutaneous malignant melanoma and was obtained from the American Type Culture Collection (ATCC^®^CRL-1619TM) and kindly provided by Dra. Marta Viana-Pereira (Life and Health Research Institute, University of Minho, Braga, Portugal). The A375 cell line was grown in Dulbecco’s modified Eagle’s medium (DMEM, PAN-Biotech, Aidenbach, Germany), supplemented with 10% FBS (PAN-Biotech) and incubated at 37 °C in a humidified environment with 5% CO_2_.

### 4.6. Cell Viability Assay

To determine the effect of the G18.EE and its fraction, G18.EE-*n*-BuOH, on A375 cell viability, the SRB (TOX-6, Sigma-Aldrich, St. Louis, MO, USA) assay was used as described previously [43]. Briefly, the cells were plated at a concentration of 25 × 10^4^ cells/mL and left overnight in a complete medium to adhere. On the following day, they were subjected to serum starvation for 2 h. After the addition of the treatments (G18.EE and G18.EE-*n*-BuOH) at different concentrations (10 to 50 µg/mL), the plates were left for 72 h at 37 °C and 5% CO_2_. DMSO (Honeywell) (control) final concentration was 0.1% in all the wells. Cells were fixed to the plate with 100 µL of TCA (10%, Sigma-Aldrich) for 1 h at 4 °C. The next step was the addition of SRB (0.4% SRB in 0.1% acetic acid), which was left to react for 30 min at room temperature (RT). To solubilise the dye, Tris base (PanReac AppliChem ITW Reagents) was added and the plates were incubated for 10 min at RT. The absorbance was measured at 490 nm (Thermo Scientific Varioskan Flash) and IC_50_ values were calculated using GraphPad Software Version 8.0.

To determine the effect of the G18.EE-*n*-BuOH subfractions on A375 cell viability, the SRB assay was also used. The methodology was the same as described above, with only one concentration (25 µg/mL, intermediate value between IC_50_ obtained for both G18.EE and G18.EE-*n*-BuOH) tested for all of the 17 subfractions (A to Q). DMSO was used as a control.

### 4.7. Statistical Analysis

Each assay was performed in triplicate and repeated at least three times independently (n ≥ 3), and results were expressed as mean ± standard deviation (SD). For IC_50_ determination, GraphPad Prism 8.0 software was used for logarithmic transformation after applying a non-linear sigmoidal dose–response regression. One-way analysis of variance (ANOVA) was used for the comparison between different conditions. The threshold used for statistical significance was *p* < 0.05.

## 5. Conclusions

Melanoma is the deadliest skin cancer type, partially due to the therapeutic resistance development. Propolis is a highly biologically active natural product produced by bees presenting antitumor properties. The ethanol extract of propolis from Gerês collected in 2018 (G18.EE) and its *n*-BuOH fraction (G18.EE-*n*-BuOH) were effective in decreasing cell biomass of the *BRAF*-mutated A375 melanoma cell line in a dose-dependent manner. In a previous paper for our research group, the G18.EE-*n*-BuOH fraction had already shown to be the most effective in reducing the viability of A375 melanoma cells, compared to the other fractions (EtOAc, *n*-hexane and water fractions) [43]. Based on that, G18.EE-*n*-BuOH was fractionated. The cytotoxic activity of the obtained subfractions was also evaluated in melanoma and the phenolic compounds present in the most active subfractions were identified. This research has made it possible to identify which phenolic compounds, among the several found in propolis, could contribute to the Gerês propolis anti-melanoma activity and, therefore, deserve more research regarding the development of new drugs against cancer. Propolis has an extremely variable chemical composition, hence the identification of the active compounds would facilitate its use in traditional medicine. Nevertheless, further studies are necessary. For instance, it would be useful to test the compounds identified in an isolated form, test different combinations of compounds, or test the compounds in combination with standard drugs, to understand the synergisms and mechanisms of action. Another question that arises is the selectivity index of the subfractions, making it necessary, in the future, to test them on normal melanocytes and/or other normal cells to evaluate their selectivity towards cancer cells.

Even so, this study confirms that Portuguese propolis is a very promising product for application in several areas, and Gerês propolis emerges as a potential adjuvant or even an alternative to conventional therapies.

## Figures and Tables

**Table 1:** IC_50_ values of G18.EE and G18.EE-*n*-BuOH in A375 melanoma cell lines. A375 cells were treated for 72 h with 10 to 50 µg/mL of each sample. Results are presented as mean ± SD (n ≥ 3).

Sample	IC_50_ (µg/mL)
	A375
G18.EE	26.87 ± 1.73
G18.EE-*n*-BuOH	23.53 ± 1.55

**Table 2:** Chemical composition of G18.EE-*n*-BuOH and subfractions E, I, and K according to UHPLC-DAD-ESI-MS^n^ analysis.

Peaks	T_R_ (min)	λ_max_ (nm)	[M–H]^−^ *m*/*z*	Probable Compounds	Samples			
					G18.EE-*n*-BuOH	E	I	K
1	2.5	253, 368	301	Ellagic acid	+	-	-	-
2	12.2	267, 291, 334	269	Apigenin	+	-	-	+
			271	Pinobanksin	+	-	+	-
3	12.8	265, 364	285	Kaempferol	+	-	-	+
4	13.2	255, 368	315	Isorhamnetin	+	-	-	-
5	17.3	310	313	Unknown	+	-	-	+
6	21.5	268, 321	253	Chrysin	+	-	-	-
			247	Caffeic acid isoprenyl ester	+	-	-	-
7	22.2	268, 330	283	Acacetin	+	-	+	+
			247	Caffeic acid isoprenyl ester	+	-	+	+
8	23	289	255	Pinocembrin	+	+	-	-
9	23.5	263, 291	269	Galangin	+	-	-	-
10	24	263, 364	299	Kaempferide	+	-	+	-
11	24.9	293	313	Pinobanksin-3-*O*-acetate	+	+	-	-
12	25.5	325	283	Caffeic acid phenylethyl ester (CAPE)	+	-	+	+
13	29.3	308	231	*p*-Coumaric acid isoprenyl ester (isomer)	+	+	-	-
14	35.5	290	417	Methylated pinobanksin-3-*O*-phenylpropionate	+	-	-	-
15	36.2	291	341	Pinobanksin-3-*O*-butyrate or	+	+	-	+
				isobutyrate				
16	31.7	293	327	Pinobanksin-3-*O*-propionate	-	+	-	-
17	39.3	292	355	Pinobanksin-3-*O*-pentenoate or	-	+	-	-
				2-methylbutyrate				
18	18.1	354, 367	329	Quercetin-dimethyl-ether	-	-	-	+

“+” = compound detected; “-” = compound not detected.

## Data Availability

Data are contained within the article.

## Sample Availability

Samples of G18 and G18.EE ae available from the authors.
